# Supplementary material for: Structural Insights into Lactococcal Siphophage p2 Baseplate Activation Mechanism
Source: Viruses. 2020 Aug 11;12(8):878. doi: 10.3390/v12080878 (PMC7472080; doi:10.3390/v12080878)
Supplement: Supplementary file 1 [file viruses-12-00878-s001.pdf]

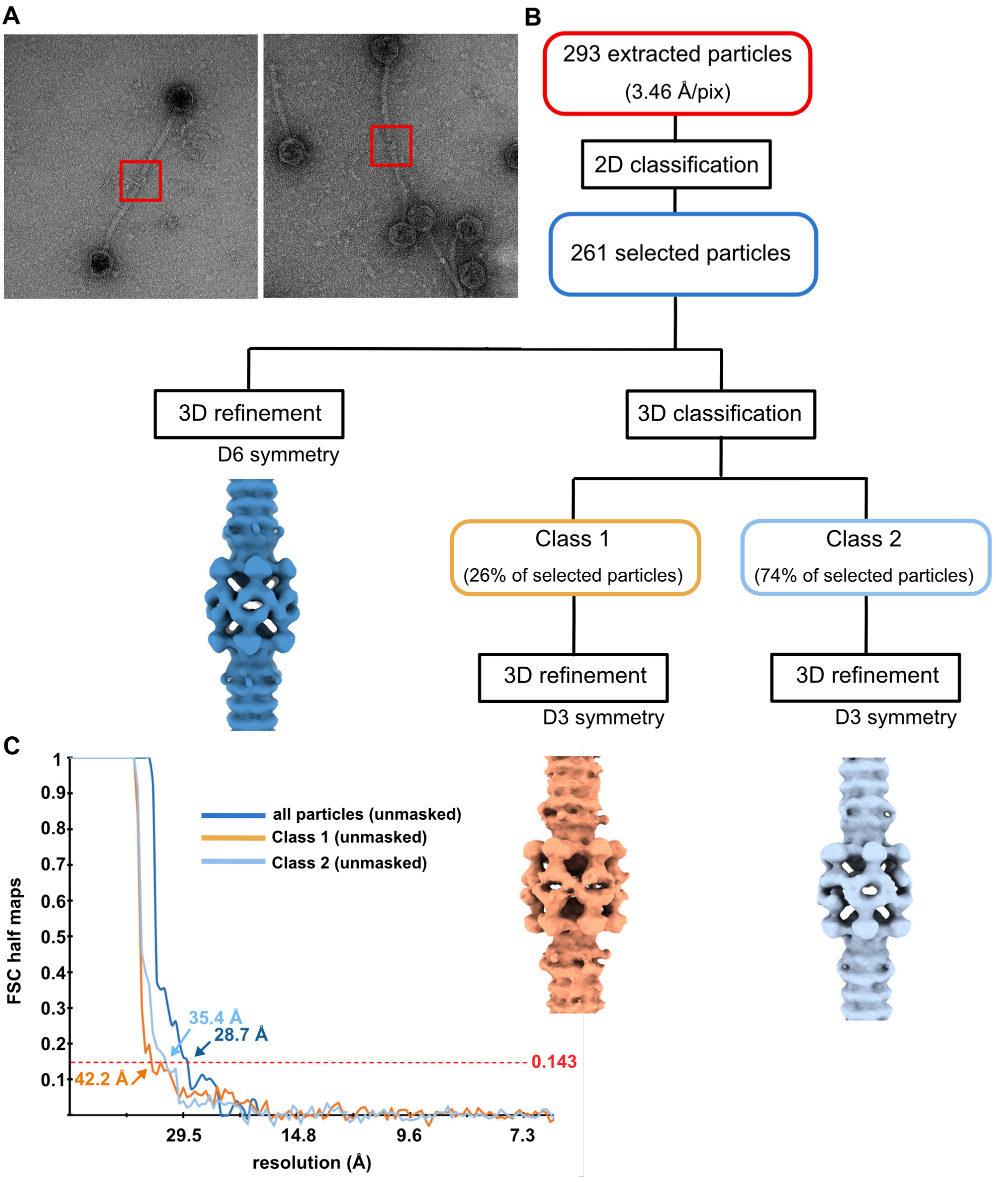


Figure S1. Negative-staining 3D reconstructions of p2 baseplate in virions complexed to VHH5. A. Representative p2 virions mixed with VHH5 observed in micrographs. The red boxes indicate the baseplate-baseplate assemblies selected for image processing. B. Data processing scheme. Selected particles, after 2D classification of extracted particles, were subjected to rounds of 3D refinement imposing D6 symmetry. Additionally, particles selected after 2D classification were subjected to 3D classification. Two 3D classes were subjected to rounds of 3D refinement imposing D3 symmetry. C. Fourier shell correlation curves between the two independently refined half-maps of final 3D reconstructions.

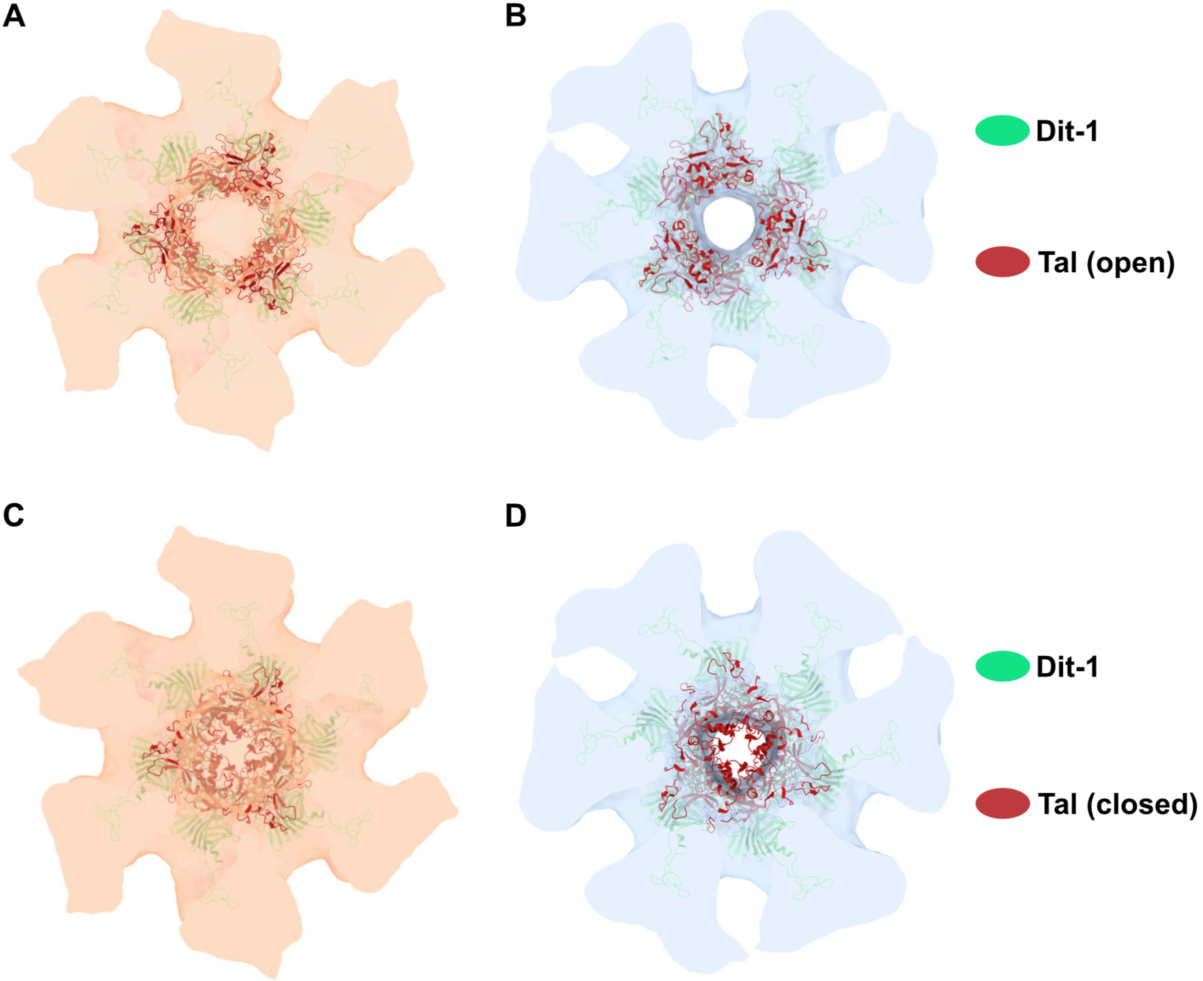


**Figure S2**. Activated baseplate in p2 virions with open and closed Tal. A-B. Ribbon representation of Dit-1 and the open Tal trimer (PDB ID 2WZP) fitted in the 3D reconstruction of the activated baseplate in p2 virions with Tal in closed (panel A) or open (panel B) conformation. C-D. Ribbon representation of Dit-1 and the closed Tal trimer (PDB ID 2X53) fitted in the 3D reconstruction of the activated baseplate in p2 virions with Tal in closed (panel C) or open (panel D) conformation.

**Table S1.** Correlation coefficients between simulated maps of molecular models rigid-body fitted into EM experimental maps (n.d: not determined, RB: rigid body)

<table>
<tr><td rowspan="4"></td><td colspan="5">Baseplate 3D reconstructions</td></tr>
<tr><td colspan="2">resting state</td><td colspan="3">activated state</td></tr>
<tr><td rowspan="2">virion<br>(EMD-1699)</td><td rowspan="2">Purified baseplate<br>(EMD-1706)</td><td colspan="3">virion + VHH5</td></tr>
<tr><td>All particles<br>(EMD-11226)</td><td>3D Class 1<br>(EMD-11225)</td><td>3D Class 2<br>(EMD-11224)</td></tr>
<tr><td colspan="6">Molecular models</td></tr>
<tr><td>RB-1: Dit-1, Tal (closed), RBP<br>(PDB ID 2WZP)</td><td>0.73</td><td>0.83</td><td rowspan="3" colspan="3">n.d</td></tr>
<tr><td>RB-1: Dit-1, Tal (closed)<br>RB-2: RBP</td><td>0.88</td><td>0.92</td></tr>
<tr><td>RB-1: Dit-1, Tal, adjusted RBP, Dit-2 with modeled galectin domain<br>(PDB ID 6ZJJ)</td><td>0.87</td><td>n.d</td></tr>
<tr><td colspan="6"></td></tr>
<tr><td>RB-1: Dit-1, Tal (open), RBP, VHH5<br>(PDB IDs 2X53, 2BSE)</td><td rowspan="2" colspan="2">n.d</td><td>0.89</td><td rowspan="2" colspan="2">n.d</td></tr>
<tr><td>RB-1: Dit-1, Tal (open), RBP, VHH5, Dit-2 with modeled galectin domain<br>(PDB ID 6ZIH)</td><td>0.9</td></tr>
<tr><td colspan="6"></td></tr>
<tr><td>RB-1: Dit-1, Tal (closed), RBP, Dit-2 with modeled galectin domain<br>(PDB ID 6ZIG)</td><td rowspan="2" colspan="2">n.d</td><td rowspan="2">n.d</td><td>0.87</td><td>n.d</td></tr>
<tr><td>RB-1: Dit-1, Tal (open), RBP, VHH5, Dit-2 with modeled galectin domain<br>(PDB ID 6ZIH)</td><td>n.d</td><td>0.88</td></tr>
</table>
